# Conjunctival lymphangioma in a 4-year-old girl revealed tuberous sclerosis complex

**DOI:** 10.3205/oc000046

**Published:** 2016-09-02

**Authors:** Florentina Joyce Freiberg, Erdmute Kunstmann, Thomas König, Juliane Matlach, Daniel Kampik

**Affiliations:** 1 Stadtspital Triemli, Department of Opthalmology, Zurich, Switzerland; 2 University Wuerzburg, Human Genetics, Wuerzburg, Germany; 3 University Wuerzburg, Department of Paediatrics, Wuerzburg, Germany; 4 University Mainz, Department of Ophthalmology, Mainz, Germany; 5 University Wuerzburg, Department of Ophthalmology, Wuerzburg, Germany

**Keywords:** tuberous sclerosis complex, conjunctival lymphangioma, ocular lymphangioma, genetic testing

## Abstract

**Background:** To present a case of conjunctival lymphangioma in a 4-year-old girl with tuberous sclerosis complex.

**Methods/results:** A 4-year-old girl presented with a relapsing cystic lesion of the bulbar conjunctiva in the right eye with string-of-pearl-like dilation of lymphatic vessels and right-sided facial swelling with mild pain. Best-corrected vision was not impaired.

Examination of the skin revealed three hypomelanotic macules and a lumbal Shagreen patch. Magnetic resonance imaging (MRI) findings displayed minimal enhancement of buccal fat on the right side. Cranial and orbital MRI showed signal enhancement in the right cortical and subcortical areas.

Genetic analysis revealed a heterozygous deletion encompassing exon 1 and 2 of the *TSC1* gene (tuberous sclerosis complex 1 gene), confirming the diagnosis of tuberous sclerosis complex.

**Conclusion:** In conjunctival lymphangioma, tuberous sclerosis complex should be considered as the primary disease.

## Introduction

Tuberous sclerosis complex (TSC) is a multisystem autosomal dominant genetic disorder caused by mutations in the genes *TSC1* (tuberous sclerosis complex 1 gene, encoding hamartin) or *TSC2* (encoding tuberin). Multiple hamartomas of the skin, heart, kidney, lung, brain, and eyes characterize TSC. Diagnosis has been based on clinical criteria recommended by a consensus conference in 1998 and 1999 (Table 1 (Tab. 1)) [1] ,[2] and is now supplemented by genetic diagnostic criteria [3].

Approximately one third of patients carry *TSC1* mutations. The *TSC1* gene consists of 23 exons, exon 1 and 2 are non-coding. The vast majority of *TSC1* lesions are point mutations, although genomic deletions of one ore more exons have been described in approximately 3% of patients [4], [5], [6], [7]. *TSC1* deletions comprising exon 1 have been shown to result in null alleles and seem to result in slightly less severe phenotypes than other mutation types in *TSC1* [7]. 

Lymphangioma was described as a vascular hamartoma of lymphatic origin [8]. These lymphangiomas present as multiple cystic lesions, a dilation of lymphatic vessels with a string-of-pearl-like appearance and may mimic allergic chemosis [9], [10]. A definite diagnosis can be made by conjunctival biopsy with immunohistochemical investigation of lymphatic vessel endothelial hyaluronan receptor-1 (LYVE 1), a membrane glycoprotein specific for lymphatic endothelium. In patients with lymphangiomas increased staining with antibody to LYVE1 is seen in immunohistochemistry [11]. 

Here we report on a 4-year-old girl with clinical diagnosis of a conjunctival lymphangioma leading to the diagnosis of tuberous sclerosis complex. To our knowledge, this is the first report of conjunctival lymphangioma in tuberous sclerosis complex.

## Case description

**Figure 1:**
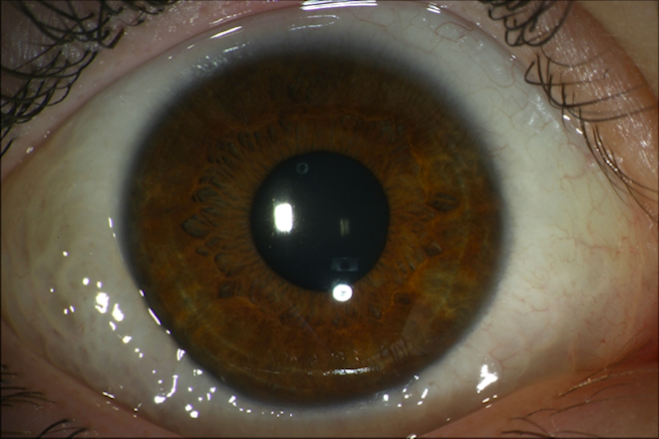
Cystic lesions of the bulbar conjunctiva in the right eye with a dilation of lymphatic vessels and a string-of-pearl-like-appearance were first noted in 2008 and persisted unchanged until last follow-up in February 2013

 In January 2008, a 4-year-old Caucasian girl presented with a relapsing cystic lesion of the bulbar conjunctiva in the right eye with a dilation of lymphatic vessels in a string-of-pearl-like appearance, a swelling of the right side of the face and mild pain in the eye. During five years of follow up no major changes in conjunctival findings were found (Figure 1 (Fig. 1)). Best-corrected visual acuity with Lea vision testing was 20/20 in both eyes. Biomicroscopy of the anterior segment, funduscopy, and intraocular pressure were normal at all times. 

### Examination

At pediatric examination, the patient claimed no acute symptoms. Physical examination and general condition was within normal limits, heart rate 92 per minute, blood pressure was 114/67mmHg, and routine blood test was unremarkable. Examination of the skin revealed three white spots (left forehead, left flank, right thigh). Wood-light did not show further depigmentation. A lumbal Shagreen patch (connective tissue nevus) was detected. Asymmetry of the face with mild swelling of the right cheek was seen. No other neurological or internal abnormalities were found. 

### Diagnostics

Magnetic resonance imaging (MRI) of the buccal area with intravenous (IV) contrast showed minimal enhancement of buccal fat on the right side without contrast enhancement. Cranial and orbital MRI with IV contrast showed a signal alteration in the right cortical and subcortical areas without contrast enhancement.

Electrocardiogram was within normal limits with an incomplete right bundle branch block. Electroencephalogram showed sharp wave complexes in variable localizations.

Echocardiography revealed an accessory left ventricular sinew thread as a variation of the norm and no relevant pathological findings.

Abdominal ultrasound showed an increase in echogenity in the left kidney, and inhomogenous parenchyma in both kidneys. No angiofibromas were found.

Genetic testing discovered a heterozygous deletion of a minimum size of 11.662 bp and a maximum size of 13.720 bp including exon 1–2 of the *TSC1* gene: chr9:g.(135820607_135819982)_(135808320_135806887)del. 

As the deletion encompasses the promoter and the transcriptional start site it is suspected to result in a functional null allele. Diagnosis of TSC was hence made clinically and confirmed by the genetic findings.

## Discussion

In this patient, the three major features of TSC were found:

- Cortical tuber (confirmed by MRI)
- Three hypomelanotic macules (“white spots”)
- Shagreen patch

TSC is a multisystem disease with the presentation of numerous hamartomas in different tissues. Lymphangioma is described as a vascular hamartoma of lymphatic origin [8]. Therefore, lymphangioma of the conjunctiva might be seen as a new clinical feature in TSC patients. In this case, conjunctival lymphangioma led to the diagnosis of TSC via cranial MRI scan. It is important to perform further interdisciplinary examinations in children with primary diagnosis of ocular lymphangioma. Roach and coworkers [1], [2] recommend neurodevelopmental testing, ophthalmic examination, electrocardiography, renal ultrasonography and cranial MRI or computed tomography (CT) to reveal major and minor features summarized in Table 1 (Tab. 1). If seizures occur, electroencephalography and a chest CT is recommended in adult women. In our case, the presumed ocular lymphangioma followed by the suggested screening tests led to the finding of three major TSC features. Biopsy of conjunctival findings was not performed because of the rare presentation of symptoms and no visible changes during 5 years of follow-up. Genetic testing confirmed the diagnosis of TSC. 

## Notes

### Acknowledgement

The authors would like to thank the patient and the patient’s family for their contribution to this case report and Mrs. K. Mayer for her support in genetic testing.

### Competing interests

All authors certify that they have NO affiliations with or involvement in any organization or entity with any financial interest (such as honoraria; educational grants; participation in speakers’ bureaus; membership, employment, consultancies, stock ownership, or other equity interest; and expert testimony or patent-licensing arrangements), or non-financial interest (such as personal or professional relationships, affiliations, knowledge or beliefs) in the subject matter or materials discussed in this manuscript.

### Patient’s consent

Patient’s consent was given to all procedures.

## Figures and Tables

**Table 1:**
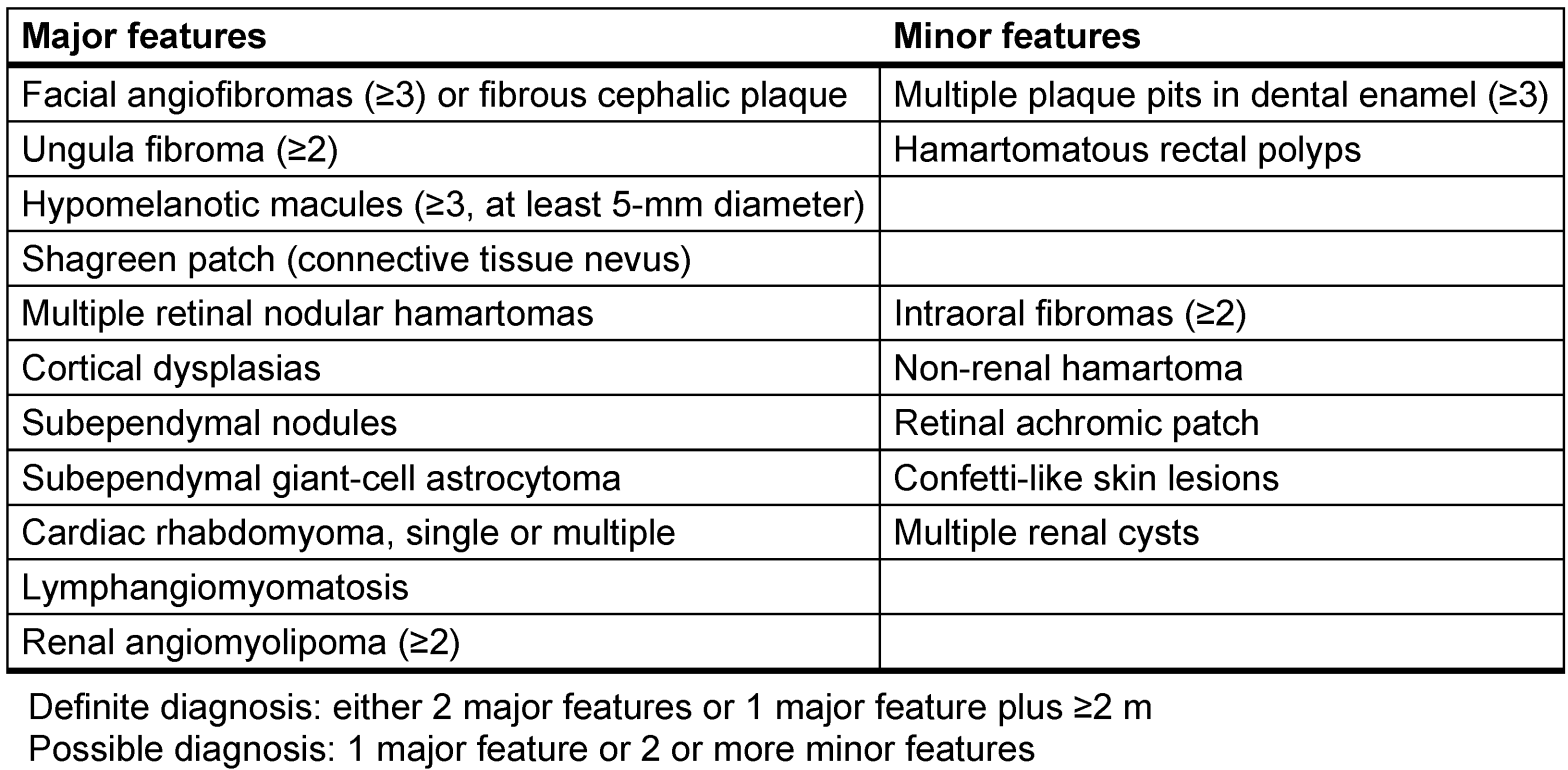
Clinical diagnostic criteria for tuberous sclerosis complex (TSC) [1], [2], [3]
